# An ectromelia virus profilin homolog interacts with cellular tropomyosin and viral A-type inclusion protein

**DOI:** 10.1186/1743-422X-4-76

**Published:** 2007-07-24

**Authors:** Christine Butler-Cole, Mary J Wagner, Melissa Da Silva, Gordon D Brown, Robert D Burke, Chris Upton

**Affiliations:** 1 Department of Biochemistry and Microbiology, University of Victoria, Victoria, BC V8W 3P6, Canada

## Abstract

**Background:**

Profilins are critical to cytoskeletal dynamics in eukaryotes; however, little is known about their viral counterparts. In this study, a poxviral profilin homolog, ectromelia virus strain Moscow gene 141 (ECTV-PH), was investigated by a variety of experimental and bioinformatics techniques to characterize its interactions with cellular and viral proteins.

**Results:**

Profilin-like proteins are encoded by all orthopoxviruses sequenced to date, and share over 90% amino acid (aa) identity. Sequence comparisons show highest similarity to mammalian type 1 profilins; however, a conserved 3 aa deletion in mammalian type 3 and poxviral profilins suggests that these homologs may be more closely related. Structural analysis shows that ECTV-PH can be successfully modelled onto both the profilin 1 crystal structure and profilin 3 homology model, though few of the surface residues thought to be required for binding actin, poly(L-proline), and PIP_2 _are conserved. Immunoprecipitation and mass spectrometry identified two proteins that interact with ECTV-PH within infected cells: alpha-tropomyosin, a 38 kDa cellular actin-binding protein, and the 84 kDa product of vaccinia virus strain Western Reserve (VACV-WR) 148, which is the truncated VACV counterpart of the orthopoxvirus A-type inclusion (ATI) protein. Western and far-western blots demonstrated that the interaction with alpha-tropomyosin is direct, and immunofluorescence experiments suggest that ECTV-PH and alpha-tropomyosin may colocalize to structures that resemble actin tails and cellular protrusions. Sequence comparisons of the poxviral ATI proteins show that although full-length orthologs are only present in cowpox and ectromelia viruses, an ~ 700 aa truncated ATI protein is conserved in over 90% of sequenced orthopoxviruses. Immunofluorescence studies indicate that ECTV-PH localizes to cytoplasmic inclusion bodies formed by both truncated and full-length versions of the viral ATI protein. Furthermore, colocalization of ECTV-PH and truncated ATI protein to protrusions from the cell surface was observed.

**Conclusion:**

These results suggest a role for ECTV-PH in intracellular transport of viral proteins or intercellular spread of the virus. Broader implications include better understanding of the virus-host relationship and mechanisms by which cells organize and control the actin cytoskeleton.

## Background

Profilins are critical to the cytoskeletal dynamics required for determination of cell shape and size, adhesion, cytokinesis, contractile force, morphogenesis and intracellular transport. Members of the profilin family of proteins are known to be key regulators of actin polymerization in eukaryotic organisms ranging from yeast to mammals, but little is known about profilin homologs found in the poxviridae and paramyxoviridae virus families [1,2].

Poxviruses are complex viruses with large double-stranded DNA genomes that encode many proteins not required for virus replication in tissue culture [3]. Some non-essential genes are involved in blocking host immune functions, while others function in pathogenesis-related pathways [4,5]. Most poxvirus genes, in fact, are not universally conserved and, as might be expected, some are found only in phylogenetically related subgroups of the poxvirus family. The poxvirus gene that encodes a homolog of cellular profilin is such a gene and appears to have been acquired by an ancestral orthopoxvirus since it is present in all fully sequenced orthopoxvirus genomes (79 to date; [6,7]), but absent from all other poxviruses. All of the poxvirus profilin homologs share 90% or greater protein sequence identity (data not shown).

Cellular profilins are believed to interact with three types of cellular molecules: actin monomers, phosphatidylinositol 4,5-bisphosphate (PIP_2_) and poly(L-proline) sequences [8]. Profilins are thought to modulate actin filament dynamics (polymerization and depolymerization) via direct binding to actin through an actin-binding domain as well as by modulation of other actin-binding proteins [9]. Over 50 proteins have been characterized as profilin ligands [8]. Numerous proteins interact with profilin directly through the poly(L-proline) binding domain, while others may bind indirectly to profilin-regulated complexes or have their activities altered by these complexes [8]. Profilins also assist in signalling between cell membrane receptors and the intracellular microfilament system by their interaction with phosphoinositides [10]. Though many of the interactions with phosphoinositides and profilin-binding proteins remain poorly understood, profilin has been implicated in diverse processes involving actin, nuclear export receptors, endocytosis regulators, Rac and Rho effectors, and putative transcription factors [8].

In contrast to its cellular homolog, the vaccinia virus profilin-homolog (VACV-PH) binds actin only weakly, has no detectable affinity for poly(L-proline), and, although it has a similar affinity for PIP_2 _[11], does not show significant binding to phosphatidyl inositol (PI) or inositol triphosphate (IP_3_) [12]. Little, therefore, is known about poxviral profilin function. However, RNA interference knockdown studies of the respiratory syncytial virus (RSV) profilin homolog showed that absence of this viral profilin had a small effect on reducing viral macromolecule synthesis and strongly inhibited maturation of progeny virions, cell fusion, and induction of stress fibers [1]. The RSV profilin homolog has been found to interact with RSV phosphoprotein P and nucleocapsid protein N. These interactions are thought to help activate viral RNA-dependent RNA polymerase [1].

Although the importance of actin filaments in poxvirus motion (and therefore cell-to-cell spread) is well understood, the specific interactions involved are not yet well-characterized [13-16]. Although viral profilin binds actin only weakly, its significant sequence similarity to cellular profilin suggested that it was a possible component in this pathway. Using the murine smallpox model, ectromelia virus, we initiated a search for proteins that interact with the ectromelia profilin homolog, ECTV-PH.

Herein we present evidence that ECTV-PH interacts with cellular α-tropomyosin and both full-length and truncated viral ATI proteins in infected cells and colocalizes to inclusion bodies and protrusions from the cells at putative actin-like tails. Many of the residues important for binding actin and other known mammalian substrates are not conserved in ECTV-PH; however, the ECTV-PH protein can be modelled onto the related structures of mammalian profilins 1 and 3.

## Results and discussion

### Sequence analysis of profilin

**Figure 1:**
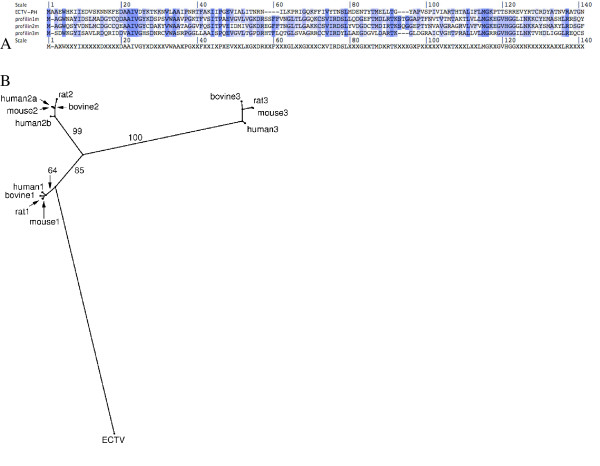
(A) T-Coffee alignment of viral and murine profilin sequences visualized with the Base-By-Base interface. Minor manual adjustments were made to the alignment based on structural analysis. Shading of individual residues indicates the degree of residue conservation between sequences (darkest = identical aa in all sequences; no shading = zero conservation). A consensus sequence is shown below the alignment. (B) Phylogenetic tree using maximum likelihood analysis for the mammalian profilin sequences available in GenBank, and ECTV-PH. The percentage bootstrap support (100 samples) is indicated along the branches.

We began our study of ECTV-PH by comparing it to various cellular profilin proteins using multiple sequence alignments. The mouse type 1 profilins appear to be most similar (~ 31% aa identity) to their orthopoxviral counterpart; mouse type 2 and type 3 profilins are ~ 25% and ~ 23% identical to the viral protein respectively (Figure 1A). An alignment of ECTV-PH and type 1, 2 and 3 profilins from mouse, human, cow, and rat showed that sequence identity conservation between each of these mammalian sequences compared to ectromelia sequence was similar to the reported percent identities for mouse profilins and ECTV-PH (within 1.5%; data not shown). Though analysis using a maximum likelihood tree (Figure 1B) supports these findings, another phylogenetic tool, maximum parsimony, places the poxviral homolog slightly closer to the type 3 profilins [2]. Although the first two methods are considered to be more reliable than maximum parsimony analysis, another piece of evidence – a shared 3-aa deletion in the viral and type 3 profilin genes – supports the maximum parsimony result. These data, apparently contradictory, could be explained by an ancestral orthopoxvirus acquiring a type 3 profilin gene from its host, and subsequent evolutionary selection leading to a slightly higher similarity with type 1 profilin.

### Structural Analysis of the ECTV-PH

**Figure 2:**
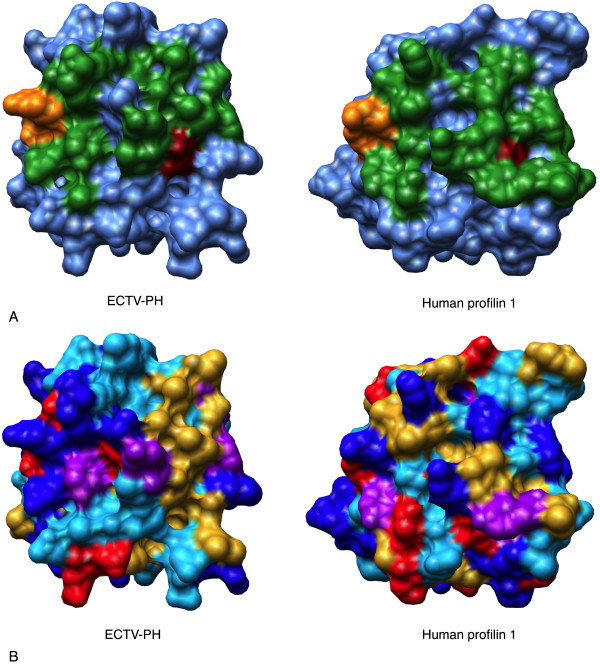
Structural comparison of the actin binding site of ECTV-PH and human profilin 1. (A) Surface diagrams of the ECTV-PH structural model and the human profilin 1 crystal structure using a blue background with the important actin binding residues coloured in green. The dark red residue represents the one residue (V-70 in ECTV-PH, V-72 in human profilin 1) that is identical between both structures; the orange residues represent two functionally conserved residues (R-120 in ECTV-PH, K-125 in human profilin 1 and D-124 in ECTV-PH, E129 in human profilin 1). (B) Surface diagrams of ECTV-PH and human profilin 1 with residues coloured by amino acid property as follows: aromatic residues (F, Y, W) in purple; negatively charged residues (D, E) in red; positively charged residues (R, H, K) in dark blue; non-polar/aliphatic residues (G, I, L, M, V) in gold; and polar/uncharged residues (N, Q, P, S, T) in light blue.

**Figure 3:**
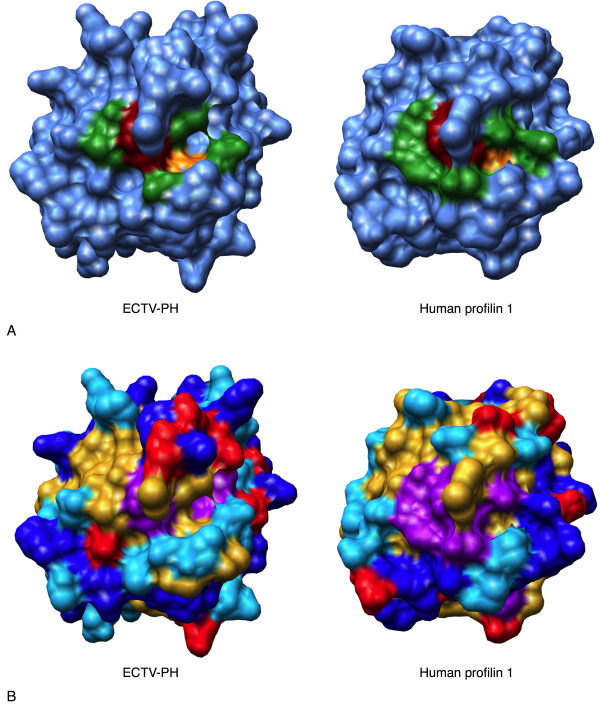
Structural comparison of the poly(L-proline) binding site of ECTV-PH and human profilin 1. (A) Surface diagrams of the ECTV-PH structural model and the human profilin 1 crystal structure using a blue background with the important poly(L-proline) binding residues coloured in green. The dark red residue represents the one residue (W-5 in ECTV-PH, W-3 in human profilin 1) that is identical between both structures; the orange residue represents the one functionally conserved residue (V-129 in ECTV-PH, L-134 in human profilin 1). (B) Surface diagrams of ECTV-PH and human profilin 1 with residues coloured by amino acid property as follows: aromatic residues (F, Y, W) in purple; negatively charged residues (D, E) in red; positively charged residues (R, H, K) in dark blue; non-polar/aliphatic residues (G, I, L, M, V) in gold; and polar/uncharged residues (N, Q, P, S, T) in light blue.

**Figure 4:**
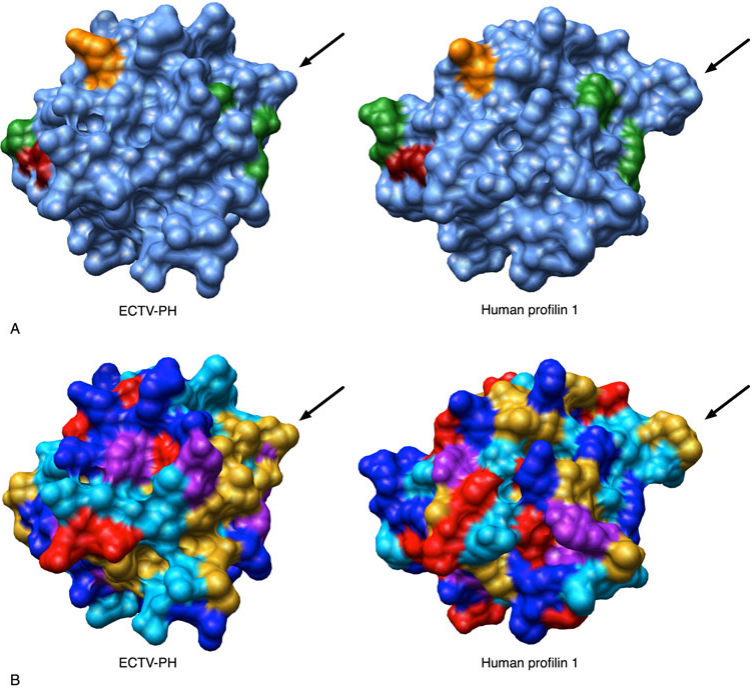
Structural comparison of the PIP_2 _binding site of ECTV-PH and human profilin 1. (A) Surface diagrams of the ECTV-PH structural model and the human profilin 1 crystal structure using a blue background with the important PIP_2 _binding residues coloured in green. The dark red residue represents the one residue (R-130 in ECTV-PH, R-135 in human profilin 1) that is identical between both structures; the orange residue represents the one functionally conserved residue (R-120 in ECTV-PH, K-125 in human profilin 1.) (B) Surface diagrams of ECTV-PH and human profilin 1 with residues coloured by amino acid property as follows: aromatic residues (F, Y, W) in purple; negatively charged residues (D, E) in red; positively charged residues (R, H, K) in dark blue; non-polar/aliphatic residues (G, I, L, M, V) in gold; and polar/uncharged residues (N, Q, P, S, T) in light blue. The arrows in panels A and B indicate the loop located between beta-strands 5 and 6 of human profilin 1 implicated in PIP_2 _binding that is reduced in size in ECTV-PH.

To provide further insight into viral profilin function, three-dimensional structural modelling of ECTV-PH was carried out. As discussed above, the sequence data indicates that ECTV-PH is closer to human profilin 1 (31% sequence identity) than 3 (23%). However, previous work has classified ECTV-PH with profilin 3 [2]. SWISS-MODEL [17] was used to model the structures of both ECTV-PH and human profilin 3 (NP_001025057.1), and each of these structures was subsequently compared by superposition to the crystal structure of human profilin 1 (PDB ID: 1FIL) [18]. We chose to show all comparisons to human profilin 1 since it is the only one of the three proteins that has a crystal structure in the PDB database (Figures 2, 3, 4). According to the root mean square deviation (RMSD) values (Table 1), ECTV-PH is closest to human profilin 3 with an RMSD value of 0.500 over 132 atoms; however, the RMSD value for human profilin 1 is 0.551 over 132 atoms and, therefore, cannot be ruled out as the closest homolog of ECTV-PH. The structure of ECTV-PH was also compared to the crystal structure of the human profilin 2b protein (1D1J chain D; [19]) as well as a homology model of human profilin 2a; however, these structures showed significantly lower structural similarity to ECTV-PH (RMSD 0.95 over 130 atoms; data not shown). Interestingly, the primary sequence of type 2 profilins is ~ 62% identical to type 1 profilins and ~ 40% identical to type 3 profilins, yet the structural similarity is relatively low (RMSD ~ 0.99 and ~ 0.98, respectively). When profilin 1 and 3 are compared they have a similar % identity (~ 43%) but much greater structural similarity (RMSD ~ 0.41). The structure of ECTV-PH was also modelled using the Robetta protein structure prediction server [20-22] and was found to be nearly identical in structure to the model created by SWISS-MODEL. Slight differences in the 3-dimensional spatial locations of two loop regions were the only differences observed between the Robetta and SWISS-MODEL models of ECTV-PH (data not shown).

**Table 1:** Root mean square deviation (RMSD) values for the superposition of human profilin 1 with ECTV-PH and human profilin 3. The right-most column gives the number of atoms over which the superposition was made

**Structure 1**	**Structure 2**	**RMSD**	**Number of atoms**
Human profilin 1	ECTV-PH	0.551	132
Human profilin 3	ECTV-PH	0.5	132
Human profilin 1	Human profilin 3	0.411	136

Human profilin contains 3 major binding domains for actin, poly(L-proline), and phosphatidylinositol 4,5-bisphosphate (PIP_2_). While the overall tertiary structure of ECTV-PH is highly conserved compared to both human profilins 1 and 3, with the closest relationship to human profilin 3, the amino acids comprising the binding regions on ECTV-PH are almost entirely different. ECTV-PH has been previously observed to have a low affinity for both actin and poly(L-proline) compared to human profilin 1 [11]. Our structural analysis supports this observation as most amino acids critical for binding in human profilin are not conserved in ECTV-PH in terms of identity or function; 20 of the 21 residues important for actin binding and 5 of the 6 important for poly(L-proline) binding in both human profilin 1 and 3 are not conserved in ECTV-PH (Table 2). Figures 2 and 3 illustrate this lack of conservation; known human profilin 1 binding residues for actin and poly(L-proline) are shown in green, while the one identical residue shared with ECTV-PH in each case appears in red. Orange represents functionally conserved residues, two for actin binding and one for poly(L-proline) binding.

**Table 2:** Comparison of residues important in actin, poly(L-proline) and PIP_2 _binding in human profilin 1, human profilin 3 and ECTV-PH. Identical and functionally conserved residues are indicated with an asterisk

**Residue in human profilin 1**	**Equivalent residue in human profilin 3**	**Equivalent residue in ECTV-PH**	**Function in human profilin 1**
W3	W4	W5	Poly(L-proline) binding*
Y6	Y7	I8	Poly(L-proline) binding
W31	W32	L33	Poly(L-proline) binding
Y59	L60	-	Actin binding
V60	Q61	-	Actin binding
N61	A62	-	Actin binding
K69	R70	F67	Actin and PIP_2 _binding
S71	C72	I69	Actin binding
V72	V73	V70	Actin binding*
I73	I74	Y71	Actin binding
R74	R75	T72	Actin binding
E82	D83	T80	Actin binding
R88	R89	L86	Actin and PIP_2 _binding
K90	K91	G88	Actin and PIP_2 _binding
T97	A95	V92	Actin binding
N99	A97	P94	Actin binding
V118	V116	T113	Actin binding
H119	H117	S114	Actin binding
G121	G119	R116	Actin binding
L122	I120	E117	Actin binding
N124	N122	Y119	Actin binding
K125	K123	R120	Actin and PIP_2 _binding*
Y128	H126	R123	Actin binding
E129	E127	D124	Actin binding*
H133	G131	N128	Poly(L-proline) binding
L134	L132	V129	Poly(L-proline) binding*
R135	R133	R130	PIP_2 _binding*
R136	M134	A131	PIP_2 _binding
Y139	A137	N134	Poly(L-proline) binding

Comparisons with human profilin regarding PIP_2 _binding are more difficult. A range of binding affinities has been reported for human profilin 1 (0.13 μM < K_d _< 35 μM) depending on the experimental method used [2,10,23]. Most recently, a dissociation constant of 985 μM was obtained using a relatively more biologically relevant assay that employed sub-micellar concentrations of PIP_2 _[10]. Because of this uncertainty in the literature, it is difficult to quantitatively compare the affinities of ECTV-PH and human profilin 1 for PIP_2_. Of the 6 amino acids important for PIP_2 _binding in human profilin 1 and 3, 5 residues are not conserved in ECTV-PH (Figure 4, Table 2) suggesting that it should have little or no binding affinity to PIP_2_. Given that Machesky observed a significant binding affinity of ECTV-PH for PIP_2_, (K_d _= 1.3 μM) [11], it is probable that nearby residues contribute to PIP_2 _binding. The loop located between beta-strands 5 and 6 of human profilin 1 has been weakly implicated in PIP_2 _binding [24], and is substantially smaller in ECTV-PH (Figure 4). It has previously been suggested that a smaller, less obtrusive loop could contribute to a lower binding affinity to PIP_2 _[24], and the observed data would seem to fit this hypothesis.

Thus, despite low sequence similarity and lack of conserved binding residues for actin, poly(L-proline), and PIP_2_, a relatively high level of structural similarity between viral and mammalian profilin is maintained. Further studies may show this structural conservation reflects functional conservation or, alternatively, adaptation of a stable protein structure by the virus for new functionality.

### ECTV-PH-interacting proteins

**Figure 5:**
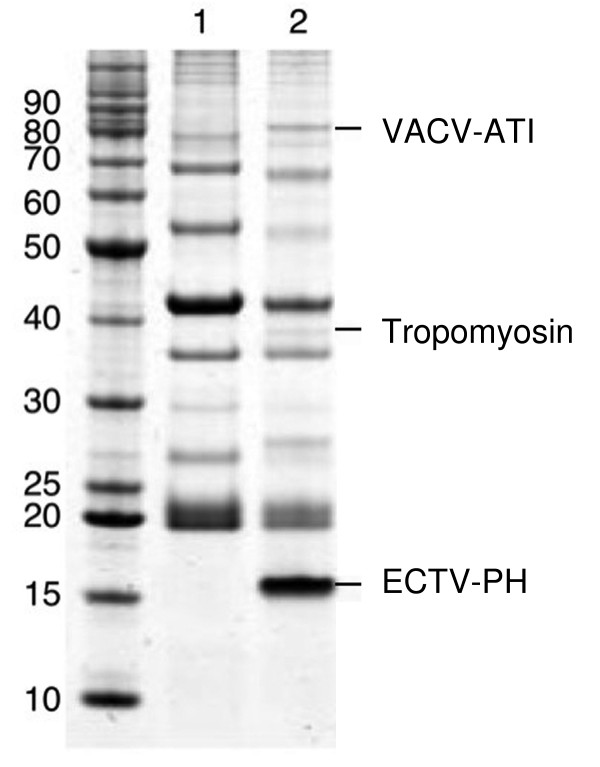
Coimmunoprecipitation of proteins that interact with ECTV-PH. His-tagged ECTV-PH was immunoprecipitated with mouse monoclonal anti-His antibodies and Protein G-Plus agarose along with any bound proteins from a BS-C-1 cell lysate. Proteins were separated by SDS-PAGE and stained with Coomassie blue. Lane 1, control immunoprecipitation; lysate contained no His-tagged ECTV-PH. Lane 2, proteins isolated from immunoprecipitation on cells expressing His-tagged ECTV-PH. Three bands at 16 kDa, 38 kDa and 84 kDa were excised from the gel and identified by mass spectrometry as indicated; a fourth band at 28 kDa was identified as a dimer of ECTV-PH in a western blot.

The first experimental step utilized immunoprecipitations to identify proteins interacting with ECTV-PH in tissue culture cells. BS-C-1 cells were infected with a recombinant VACV strain WR vTF7-3 expressing a T7 polymerase, and then transfected with a plasmid containing the gene of interest, a Histidine (His)-tagged ECTV-PH. Late in infection (after 16 h), proteins were extracted from the cell and subjected to a penta-His antibody to selectively precipitate ECTV-PH and any associated proteins. Interacting proteins were affinity captured on Protein-G agarose and subjected to SDS-PAGE analysis. The resulting gel is presented in Figure 5. Lane 1 shows a negative control using cell lysate containing no ECTV-PH (bands are proteins that interact non-specifically with precipitating agents.) Four bands appear in lane 2 that are not present in lane 1; of these, the 16 kDa and 28 kDa bands are unbound ECTV-PH monomers and dimers, respectively. We have shown by western blotting that this protein can maintain a dimerized form, despite the denaturing conditions of an SDS-PAGE gel (data not shown). The 38 and 84 kDa bands are proteins that interact with ECTV-PH. These were excised from the gel and identified via mass spectrometry as VACV-WR 148, an 84 kDa protein which belongs to the orthopoxvirus A-type inclusion (ATI) protein family (protein accession no. AA089427.1), and α-tropomyosin, a 38 kDa cellular actin-binding protein (protein accession no. AAA61226). Although lane 2 is slightly under-loaded relative to lane 1, the non-specific interacting proteins are generally comparable between the two lanes. It is interesting, however, that some of the proteins appear to migrate slightly faster in lane 2; this may represent differential protein processing in virus infected cells.

**Figure 6:**
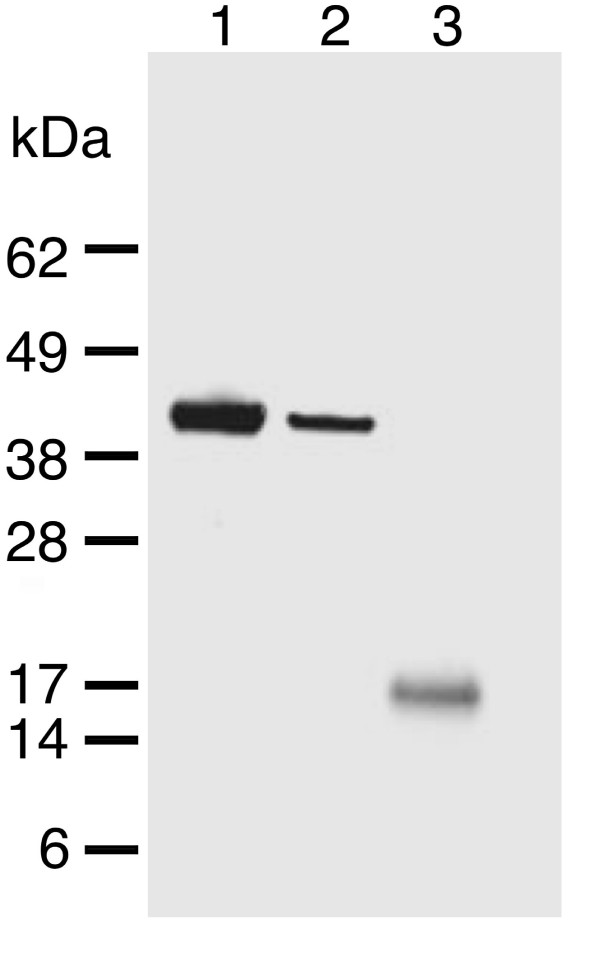
Western blot to test for actin in coimmunoprecipitates using rabbit IgG anti-actin primary antibody. Lane 1, purified rabbit muscle actin showing an immunoreactive band at 42 kDa (positive control). Lane 2, starting cell lysate showing presence of actin. Lane 3, proteins that coimmunoprecipitated with ECTV-PH as described in Figure 2 showing absence of detectable actin.

As tropomyosin is an actin-binding protein and viral profilin is known to bind actin (though weakly in the case of viral profilin [11]), two additional investigations were performed that demonstrate the tropomyosin-profilin interaction is direct. Firstly, a western blot of the immunoprecipitated ECTV-PH sample with a polyclonal anti-actin primary antibody failed to detect actin (Figure 6). Lanes 1 and 2 contain purified rabbit muscle actin and starting cell lysate from which ECTV-PH and ECTV-PH-interacting proteins were isolated, respectively. Strong immunoreactive bands at 42 kDa are observed in both lanes, indicating significant levels of actin in the initial cell lysate. Lane 3 contains proteins that coimmunoprecipitated with ECTV-PH; no immunoreactive band at 42 kDa indicates that if actin is present, levels are below the detection threshold of the western blot. These data agree with findings (discussed earlier) that the viral profilin homolog has a low binding affinity for actin [9]. The immunoreactive band at 17 kDa corresponds to the MW of Protein G (precipitating agent), indicating that it retains some capacity to bind to antibodies even after separation by SDS-PAGE and transfer to blotting membrane.

**Figure 7:**
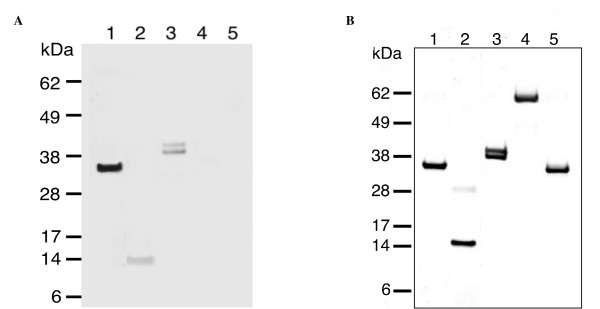
Far western blot probed with tropomyosin and detected with mouse anti-tropomyosin primary antibodies. (A) Lane 1, purified porcine muscle tropomyosin showing an immunoreactive band at 37 kDa (positive control). Lane 2, purified ECTV-PH; the immunoreactive band at 15 kDa represents its interaction with tropomyosin. Lane 3, purified rabbit muscle actin; immunoreactive bands at 42 kDa and 43 kDa represent tropomyosin interaction with different actin isoforms. Lanes 4 and 5 contain His-tagged RelA and His-tagged bovine serum albumin respectively (negative controls); no immunoreactive bands are present. (B) Corresponding SDS-PAGE stained with Coomassie blue. Lane assignments are as described in (A).

Another possibility was that interaction occurred through this His-tag on the ECTV-PH protein. A far-western blot was performed, using nickel-column purified ECTV-PH probed with porcine muscle tropomyosin protein and detected with mouse monoclonal anti-tropomyosin IgG_1 _primary antibody (Figure 7, lane 2). Since no tropomyosin bound to a His-tagged control protein or BSA, we concluded that the interaction is not due to the His-tag (Figure 7, lanes 4 and 5).

### Sequence analysis of viral A-type inclusion proteins

The next step was to investigate the poxviral ATI proteins that interact with ECTV-PH. The majority of orthopoxviruses encode an ATI protein that is expressed late in infection at approximately the same time as the profilin homolog [25]. ATI proteins are present either as a full-length protein, found in cowpox virus (CPXV) and ECTV, or a truncated form of the protein found in most other orthopoxviruses. Full-length ATI proteins form large bodies in the cytoplasm that contain intracellular mature virions (IMV), and are thought to be important in survival and dissemination of the virions [26,27]. Although the function of truncated ATI proteins is poorly understood, in VACV they do associate with mature virions [26], and the conservation of these truncated genes suggests the protein does confer an advantage to the virus during its life cycle.

**Figure 8:**
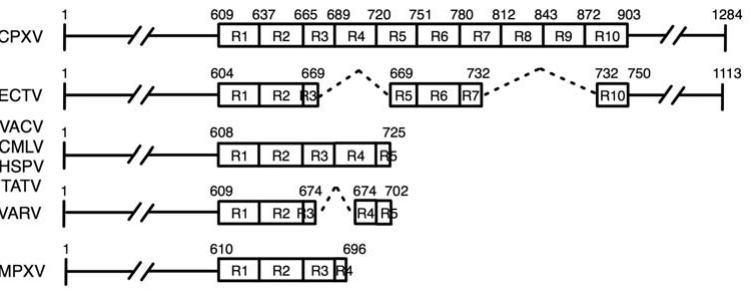
Schematic of Poxviral ATI proteins shown in groups with similar sequences. Virus groups are abbreviated as follows: Cowpox virus (CPXV), Ectromelia virus (ECTV), Vaccinia virus (VACV), Horsepox virus (HSPV), Camelpox virus (CMLV), Taterapox virus (TATV), Variola virus (VARV), and Monkeypox virus (MPXV). Numbers indicate aa positions. Ten tandem repeats, as described by Osterrieder *et al*. [29], are represented by boxes labelled R1 through R10. Deletions are indicated by broken triangular lines. Double-backslashes indicate sequence not shown in the N- and C- terminal regions.

ATI proteins are present in over 90% of the orthopoxvirus genomes sequenced to date (66 out of 73 total). Interestingly, the CPXV and ECTV ATI proteins are approximately 60% longer than the highly conserved truncated version. The longest ATI protein, 1284 aa in length, is encoded by CPXV strain Brighton Red. The first ~ 600 residues, conserved in the truncated version, are followed by a series of 10 tandem peptide repeats, each 24–32 aa long, and a carboxyl (C)-terminal region of ~ 380 residues (Figure 8). Several of these repeats are absent in ECTV-ATI; other orthopoxviruses have larger deletions in the repeat region as well as in the C-terminus. The ATI gene is completely absent from several monkeypox and VACV genomes, and is, therefore, not essential to virus replication. However, the widespread conservation of the truncated portion indicates that this gene likely encodes a beneficial and selectable trait.

### Localization of the ECTV-PH and VACV-WR A-type inclusion proteins in infected cells

**Figure 9:**
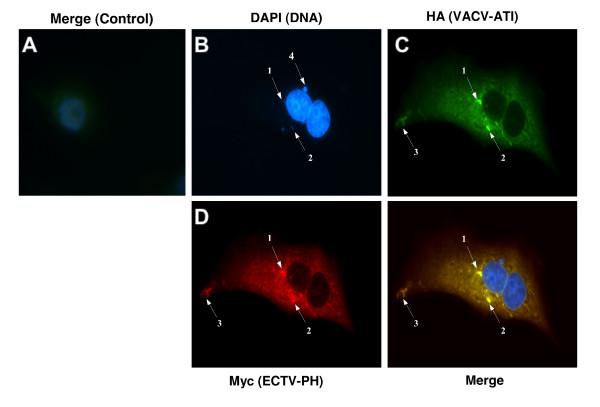
Investigation of colocalization of ECTV-PH and VACV-ATI by immunofluorescence. HA-tagged VACV-ATI protein, a truncated-type ATI protein, and Myc-tagged ECTV-PH were overexpressed in BS-C-1 cells using a vTF7-3 transient expression system. (A) Control cells, infected with vTF7-3 and transfected with calf thymus DNA, show DAPI staining of cellular nuclei and little background staining with anti-HA and anti-Myc antibodies (negative control). (B) DAPI staining of cellular nuclei and viral DNA. Discrete areas of DNA in the cytoplasm are indicated by arrows 1, 2 and 4. (C) VACV-ATI protein (green) and (D) ECTV-PH (red) are both distributed throughout the cytoplasm. Arrows 1, 2 and 3 indicate areas of high protein colocalization. (E) Merged view of panels (B-D). Arrows 1 and 2 indicate putative inclusion bodies where VACV-ATI, ECTV-PH, and viral DNA colocalize. Arrow 3 indicates the colocalization of VACV-ATI and ECTV-PH to a putative protrusion from the cell surface.

To determine if ECTV-PH and VACV-WR 148 (a truncated ATI protein) colocalize in poxvirus-infected cells, hemagglutinin (HA)-tagged VACV-WR 148 (VACV-ATI) and Myc-tagged ECTV-PH were co-expressed with the vTF7-3 transient expression system and visualized by indirect immunofluorescence. Since anti-His antibodies are known to cross-react with cellular proteins, a Myc-tagged ECTV-PH expression plasmid was constructed for use with the vTF7-3 virus in place of the His-ECTV-PH. A mock-infected control cell stained with both anti-HA and anti-Myc antibodies as well as 4'6-diamidino-2-phenylindole (DAPI) DNA staining is shown in Figure 9A; very little background antibody binding is seen. Figure 9B shows an infected cell subjected to DAPI staining (blue fluorescence indicates the nucleus). In the infected cell, both VACV-ATI (Figure 9C; green fluorescence) and ECTV-PH (Figure 9D; red fluorescence) are visible throughout the cytoplasm. However, several regions have brighter immunofluorescence signals for both proteins; the *merge *view suggests that these are sites of colocalization (Figure 9E arrows 1–3). Truncated ATI proteins have been observed to aggregate and form small, irregularly-shaped, unstable inclusion bodies [28]. The morphology of the putative regions of colocalization in Figure 9E (arrows 1 and 2) matches this description. Two extranuclear regions stained for DNA (Figure 9B arrows 1 and 2) overlap with the observed bodies. If these are indeed unstable inclusion bodies formed by aggregated truncated ATI proteins, this evidence suggests that they are still able to sequester intracellular mature virions (IMV). In contrast, the viral factory indicated by arrow 4, a discrete area in the cytoplasm containing actively replicating viral DNA, does not colocalize to the putative inclusion bodies. Finally, ECTV-PH and VACV-ATI also appear to colocalize to a structure near the cell periphery (Figure 9E arrow 3), resembling protrusions from the cell surface induced by cell-associated virions (CEV) during infection.

### Localization of the ECTV-PH and ECTV-Moscow A-type inclusion proteins in infected cells

**Figure 10:**
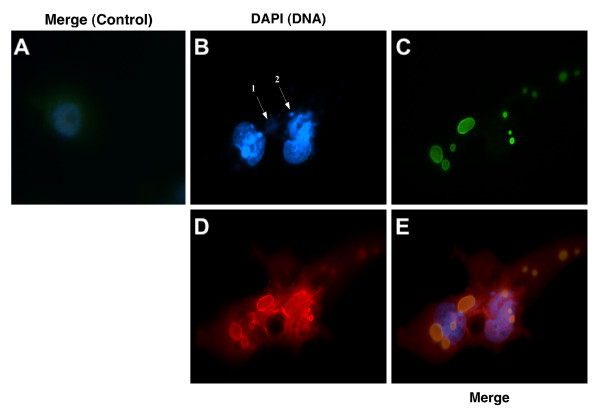
Investigation of colocalization of ECTV-PH and ECTV-ATI by immunofluorescence. HA-tagged ECTV-ATI protein, a full-length type ATI protein, and Myc-tagged ECTV-PH were overexpressed in virus-infected BS-C-1 cells using a vTF7-3 transient expression system. (A) Control cells, infected with vTF7-3 and transfected with calf thymus DNA, show DAPI staining of cellular nuclei and little background staining with anti-HA and anti-Myc antibodies (negative control). (B) DAPI staining of cellular nuclei and viral DNA. Discrete areas of DNA in the cytoplasm are indicated by arrows 1 and 2. (C) ECTV-ATI (green) is present only in discrete areas (large inclusion bodies) located in the cytoplasm. (D) ECTV-PH (red) partially localizes to inclusion bodies as well as being partially distributed throughout the cytoplasm. (E) Merged view of panels (B-D) shows localization of ECTV-PH and ECTV-ATI, but not viral DNA, to inclusion bodies.

To characterize the interaction between ECTV-PH and full-length poxvirus ATI proteins, Myc-tagged ECTV-PH and HA-tagged ECTV-Moscow-128 A-type inclusion (ECTV-ATI) proteins were overexpressed and localized in BS-C-1 cells using the same vTF-3 transient expression system and antibodies as previously described. ECTV-ATI has been previously shown to form large, round inclusion bodies in the cytoplasm of the host cell [29]. These bodies are clearly visible in Figure 10C. Unlike VACV-ATI, the ECTV-ATI protein appears to be completely localized to these inclusions in the cytoplasm, which are excluded from the nucleus as seen by DAPI staining (Figure 10B). This complete localization to the inclusion bodies supports earlier findings that ATI proteins are associated only with IMVs [19]. Though ECTV-PH also largely colocalizes to these inclusion bodies (Figure 10D), some of the protein remains distributed throughout the cytoplasm. This suggests that ECTV-PH may also interact with other proteins in the cytoplasm, such as cellular tropomyosin (as previously demonstrated in Figures 5 and 7). Viral DNA (Figure 10B arrows 1 and 2) does not appear to localize to the inclusion bodies.

Taken together, the results of these two immunofluorescence experiments suggest that ECTV-PH localizes to inclusion bodies formed by both truncated and full-length versions of the viral ATI protein in the cytoplasm of the host cell. As the amino (N) terminus and first two tandem repeats are the only domains these proteins share, it is reasonable to conclude that this shared region contains the site of interaction with the profilin homolog. In addition, the colocalization of viral profilin and truncated ATI protein to protrusions from the cell surface suggests that these proteins may together be involved in intercellular transport of the virus.

### Localization of the ECTV-PH and cellular tropomyosin proteins in infected cells

The role of tropomyosin is well understood in skeletal muscle, where it regulates the actin-myosin interaction, controlling muscle contraction. However, the role of tropomyosin in the cytoskeleton has remained elusive. Actin filaments vary in composition due to utilization of distinct isoforms of both actin and tropomyosin, which are temporally and spatially regulated [30]. It has been demonstrated that tropomyosin isoforms differentially regulate actin filament function and stability [30]. As ECTV-PH binds tropomyosin and may be involved in actin polymerization, we investigated the localization of ECTV-PH and cellular tropomyosin in poxvirus-infected cells using indirect immunofluorescence.

**Figure 11:**
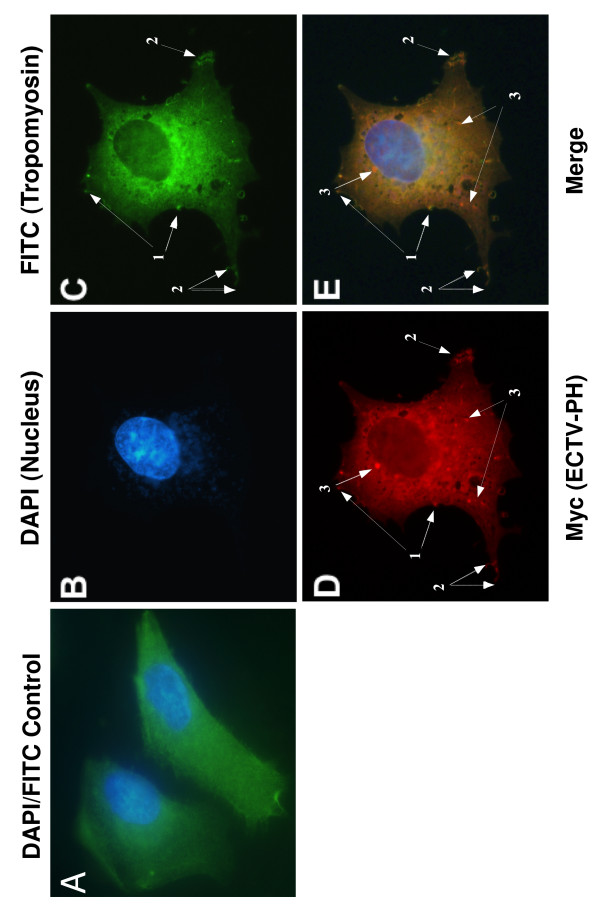
Investigation of colocalization of ECTV-PH and cellular tropomyosin by immunofluorescence. Myc-tagged ECTV-PH was overexpressed in virus-infected BS-C-1 cells using a vTF7-3 transient expression system. (A) Mock-infected control cells stained with anti-tropomyosin antibodies and visualized with FITC (green) show a relatively uniform distribution of endogenous tropomyosin throughout the cytoplasm. DAPI staining (blue) shows the cellular nuclei. See Figure 9A for the corresponding anti-Myc control. (B) DAPI staining shows the cellular nucleus and viral DNA (blue). (C) Endogenous tropomyosin (green), and (D) ECTV-PH (red) are both distributed throughout the cytoplasm but colocalize to structures resembling actin tails (arrows labelled 1) and to protrusions from the cell surface (arrows labelled 2). ECTV-PH also localizes in high concentrations to structures resembling inclusion bodies formed by truncated ATI proteins (presumably from the recombinant vaccinia used to infect the cells (arrows labelled 3)). (E) Merged view of panels (B-D) showing colocalization of tropomyosin and ECTV-PH to structures at the cell periphery as described above, indicated by arrows 1 and 2. Arrows labelled 3 indicate putative inclusion bodies, as described above.

Endogenous cellular tropomyosin was relatively uniformly distributed throughout the cytoplasm in the mock-infected control cells (Figure 11A; green fluorescence). In the infected cell, both tropomyosin (11C, green fluorescence) and ECTV-PH (11D, red fluorescence) were also observed throughout the cytoplasm. Neither is present in the nucleus, as is shown by DAPI DNA staining (11B; blue fluorescence). It is possible that tropomyosin and ECTV-PH interact with each other in the cytoplasm and/or with different cytoplasmic proteins, though due to the widespread distribution of both, no definite conclusions are possible.

Intriguingly, some ECTV-PH and the endogenous cellular tropomyosin appear to colocalize in higher concentrations to structures resembling actin tails (Figure 11E, arrows labelled 1); these are known to support extracellular enveloped virus (EEV)-containing protrusions from the cell surface (Figure 11E, arrows labelled 2) that are important for the intercellular spread of poxviruses [31]. ECTV-PH (but not tropomyosin) also localizes in high concentrations to structures resembling inclusion bodies (arrows labelled 3). These are presumably aggregates of the truncated ATI protein encoded by vTF7-3, the recombinant vaccinia virus used in the transient expression system. Though similar to the inclusion bodies formed when the truncated VACV-ATI is overexpressed (Figure 9E arrows 1 and 2), those seen here are more spherical, suggesting that overexpression of the protein may affect the morphology of the putative inclusion bodies.

### Summary of the Immunofluorescence Results

Our immunofluorescence results show that full-length ECTV-ATI and ECTV-PH colocalize to inclusion bodies, where IMVs are known to be sequestered [27]. Truncated ATI proteins do not form stable inclusion bodies, and the structures formed are seen to be small and irregularly shaped in our study in agreement with previous work [28], yet we observed some colocalization of ECTV-PH and VACV-ATI proteins to putative inclusion bodies and protrusions on the cell surface. IMV particles have been shown to travel along microtubules and form intracellular enveloped virus (IEV) particles that then travel to the cell surface [15]. Our results suggest that profilin may be involved with inclusion bodies and IMV transport. Though the immunofluorescence data for tropomyosin are less conclusive, it is possible that tropomyosin and ECTV-PH are also involved in release and/or intercellular transport of viral particles. Because ECTV-PH was overexpressed using a T7 promoter, it is possible that the protein was more widely distributed than when it is expressed at endogenous levels, in effect weakening the visualization of concentrated ECTV-PH and making interpretation of results more difficult.

In a previous study by Blasco et al., cells infected with a deletion mutant of VACV-WR lacking the profilin homolog showed normal plaque formation, infectivity, and IMV/EEV production, movement, and release [32]. This study also showed by fluorescence microscopy that viral profilin does not associate with actin filaments within the infected cell. In agreement with this, we did not detect an interaction between actin and ECTV-PH. However, we did observe associations between ECTV-PH, tropomyosin and ATI proteins at cellular protrusions and putative actin tails. It is possible that VACV profilin used in the Blasco study has functional differences from ECTV-PH used in our study. It would be interesting to perform a similar deletion study with ECTV-PH and visualize viral particle movement using confocal microscopy or immuno-electron microscopy. Although the profilin protein sequences are 90% identical between VACV and ECTV, many genes in the large poxvirus genome are different, including the ATI protein that is full-length in ECTV and truncated in VACV. Another possibility is that VACV profilin is more connected to processes involving PIP_2 _than those involving actin (proposed by Machesky et al. and Blasco et al. [11,32]). Our structural analysis (discussed earlier) does not dismiss this possibility, though most of the PIP_2 _binding residues in the active site are not conserved. Since both the Blasco study and our study were done in cell culture, it is possible that during the natural infection of the host, the associations we observed between ECTV-PH, tropomyosin and ATI protein may become important in actin-associated events. Although not necessarily required for viral particle production and movement, profilin may have a role to play when present in this context.

Taken as a whole, the immunofluorescence results support the idea that ECTV-PH may have some role in intracellular transport of viral proteins in the cytoplasm or intercellular spread of the virus. However, further studies are needed to demonstrate these functions, as well as confirm the colocalization of ECTV and tropomyosin within the cell. Subsequent immunofluorescence studies examining the association of ECTV-PH, tropomyosin, and ATI proteins with viral membrane proteins and actin, and examining the movement of these proteins in infected cells would provide valuable insight.

## Conclusion

In this study, we characterize a profilin homolog, ECTV-PH, encoded by ectromelia virus. Poxviruses are known to utilize the cellular cytoskeleton for the transport of virions and viral components during viral infection, although the specific mechanisms are not well understood. The ability of cellular profilin to bind directly to actin and to modulate the activities of other actin-binding proteins makes the viral homolog a candidate for involvement in these processes.

Our investigation provides evidence that suggests viral profilin plays a role in protein or viral particle localization. We show that ECTV-PH associates with viral ATI protein in immunoprecitations of infected cells. Furthermore, immunofluorescence studies show strong colocalization of ECTV-PH with full-length and truncated ATI proteins in inclusion bodies. In the case of truncated ATI protein, colocalization with ECTV-PH also occurs at protrusions from the cell surface. The formation or utilization of these structures that are involved in the protection and spread of the virus may be facilitated by the action of the profilin homolog.

ECTV-PH also directly associates with cellular tropomyosin, an actin-binding protein and regulator of actin filament function and stability. The extent to which ECTV-PH and tropomyosin colocalize in immunoflourescence studies is less clear; however, our results raise the possibility that ECTV-PH and tropomyosin may associate at protrusions from the cell surface and putative actin tails. These data support a potential role for these proteins in intracellular transport of IMVs or viral proteins, or intercellular spread of the virus.

Three-dimensional modelling showed that although the viral profilin homolog shares only 31% and 23% amino acid identity with mammalian profilin 1 and 3 respectively, the overall structure of the proteins are very similar. The lack of conservation of residues known in human profilin to be involved in binding of actin, poly(L-proline), and PIP_2 _suggests that the function of the poxviral homolog may be quite different from cellular profilins and illustrates how genes that are hijacked by viruses may be rapidly modified and apparently new functions selected for (e.g. binding of viral ATI protein). It is interesting that although the primary sequences have diverged considerably, the structures have to a large degree been maintained. This suggests that there is an inherent value to a stable protein structure that can support a variety of functional interaction surfaces. Also, since cellular profilins are known to interact with multiple protein and phosphoinositide ligands, it is possible that the poxvirus profilin homologs have maintained some of these interactions that were not detected in our immunoprecipitation experiment. Low protein concentration or either weak or transient interactions could result in such interactions being undetected. The conservation of profilin-like genes in current orthopoxviruses (greater than 90% aa identity) indicates that these proteins perform an important function during viral infection. Further characterization of the mechanisms by which poxviruses manipulate the cytoskeleton will not only result in a deeper understanding of the virus-host relationship, but may also give a fresh insight into mechanisms by which uninfected cells organize and control the actin cytoskeleton.

## Methods

### Sequence Analysis

All poxviral protein sequences were obtained from the Viral Orthologous Clusters (VOCs) database [6,7]. Other sequences were retrieved from GenBank: human profilin 1 (NP_005013), profilin 2 isoform a (NP_444252), profilin 2 isoform b (NP_002619), and profilin 3 (NP_001025057); *Bos taurus *profilin 1 (NP_001015592), profilin 2 (Q09430), and profilin 3 (NP_001071413); *Mus musculus *profilin 1 (NP_035202), profilin 2 (NP_062283), and profilin 3 (NP_083579); *Rattus norvegicus *profilin 1 (NP_071956), profilin 2 (NP_110500), and profilin 3 (XP_001065833). Multiple sequence alignments and percent identity tables were created with Base-By-Base [33] using the T-Coffee alignment algorithm [34] with minor manual adjustments to the profilin alignment based on structural analysis.

Phylogenetic trees were constructed from 14 aligned profilin amino acid sequences using the PHYLIP package [35]. The maximum likelihood tree was calculated with the "proml" program, using the Jones-Taylor-Thornton model of amino acid substitution [36], with a constant rate of change across sites, and allowing global rearrangements. The input sequence order was randomized 5 times, and from the resulting 5 output trees, the highest-scoring tree was selected. The maximum parsimony tree (data not shown) was calculated with the "protpars" program, using ordinary parsimony, with all sites equally weighted. Input order was again randomized 5 times, and the best tree chosen from the 5 trials. Bootstrap values were created using "seqboot" to generate 100 bootstrap samples of the input sequences. For both maximum parsimony and maximum likelihood, trees were computed for all bootstrap data sets using the same parameters as the original data; the consensus trees and bootstrap values were calculated using "consense". Trees were drawn with the "drawtree" program, and edited with the Xfig Drawing Program for the X Window System [37].

### Homology modelling

The ECTV-PH primary protein sequence (NP_671660.1) was submitted to the SWISS-MODEL [17] server using the "First Approach" mode with default settings. The server identified 4 profilin proteins as having a high degree of sequence identity with ECTV-PH based on BLASTp results [38]. These 4 profilins were then used for the ECTV-PH structural model: human platelet profilin 1 (high salt; 1FIL; [18], human platelet profilin 1 (low salt; 1FIK; [18]), human profilin NMR structure (1PFL; [39]), and bovine profilin complexed with beta-actin (1HLU; [40]). The homology model of ECTV-PH was superimposed and subsequently compared to the crystal structure of human profilin 1 (1FIL) using the MatchMaker feature of the Chimera visualization software [41]. The structural model of ECTV-PH was confirmed by modelling the primary sequence of ECTV-PH using the Robetta protein structure prediction server [20-22] using the human platelet profilin crystal structure as a template (1CJF chain A; [42]).

The primary protein sequence of the human profilin 3 protein (NP_001025057.1) was also modelled with SWISS-MODEL [17] using default settings. The SWISS-MODEL server modelled the structure of human profilin 3 based on 4 profilin proteins; human platelet profilin 1 (low salt; 1FIK; [18]), human platelet profilin 1 (high salt; 1FIL; [18]), human profilin 1 NMR structure (1PFL; [39]), and human platelet profilin 1 complexed with a proline-rich ligand (1CF0; [42]). A structural model for human profilin 2a (data not shown) was created using SWISS-MODEL in the same manner as both ECTV-PH, and human profilin 3, using the following protein crystal structures as templates: human profilin 2b (1D1J; [19]) and bovine profilin complexed with beta-actin (1HLU; [40]).

### Expression and purification of recombinant proteins

The polymerase chain reaction (PCR) was utilized to amplify the target genes from ECTV-Moscow DNA and incorporate a primer sequence encoding a 6-histidine tag onto the 5' end of each gene. PCR products were cloned into the pENTR/SD/D-Topo entry vector, and then subcloned into the pDEST14 destination vector to generate expression clones as per the manufacturer's instructions (Invitrogen Life Technologies, Carlsbad, CA, USA). A recombinant VACV strain WR vTF7-3 (ATCC VR-2153; [43]) expressing a T7 polymerase was used to transiently overexpress His-tagged ECTV-PH in BS-C-1 cells. Tissue culture reagents were obtained from Gibco BRL Inc. (Gaithersburg, MD, USA). The African green monkey kidney cell line BS-C-1 (ATCC CCL 26), was grown in complete Dulbecco's modified Eagle medium supplemented with 10% newborn bovine serum, 50 U/ml penicillin, 50 μg/ml streptomycin and GlutaMAX-II (Gibco). BS-C-1 cells were lysed using a French Pressure Cell (American Instrument Company, Silver Spring, Maryland, USA) and His-tagged ECTV-PH was purified using a Ni-NTA column (Invitrogen Life Technologies) with a Bio-Rad Biologic low-pressure chromatography system and a 0–300 mM imidazole elution gradient (Bio-Rad, Richmond, CA, USA). EDTA and glycerol were added to the fractions (final concentrations of 1 mM and 10%, respectively) to prevent degradation and aggregation of the proteins, which were then stored at -20°C. Protein concentrations were determined using Bradford Reagent (Sigma-Aldrich, Oakville, ON, Canada) following manufacturer's instructions.

### Western blots

Purified protein or cellular lysates were separated by SDS-PAGE on pre-cast NuPAGE Novex 4 – 12% Bis-Tris 12 well gels (Cat # NP0322BOX, Invitrogen Life Technologies) using an Xcell SureLock Mini-cell apparatus as per the manufacturer's instructions (Invitrogen Life Technologies). The proteins were transferred to a Trans-Blot nitrocellulose membrane (Cat #12011, Bio-Rad) using a Bio-Rad mini trans-blot cell apparatus as per the manufacturer's instructions (Bio-Rad). The blot was detected with a 1:1500 dilution of primary antibody and a 1:2500 dilution of secondary antibody as follows. His-tagged proteins were detected and visualized using mouse IgG_1 _anti-penta His primary antibody (Cat # 34660, QIAGEN, Chatsworth, CA, USA) and rabbit anti-mouse IgG (H&L) IRDye 800 conjugate secondary antibody (Cat # 610432020, Rockland Immunochemicals Inc., Gilbertsville, PA, USA). Myc-tagged proteins were detected and visualized using rabbit polyclonal anti-Myc primary antibody (Cat # 2272, Cell Signalling Technology, Beverly, MA, USA) and goat anti-rabbit IgG (H&L) IRDye 800 conjugate secondary antibody (Cat # 611132122, Rockland Immunochemicals Inc.). HA-tagged proteins were detected and visualized using mouse IgG_1 _anti-HA Alexa Fluor 488 conjugate antibody (Cat # A21287, Molecular Probes Inc., Eugene, OR, USA) and rabbit anti-mouse IgG (H&L) IRDye 800 conjugate secondary antibody (Cat # 610432020, Rockland Immunochemicals Inc.). Actin was detected and visualized using rabbit IgG anti-actin primary antibody (Cat # A5060, Sigma-Aldrich) and goat anti-rabbit IgG (H&L) IRDye 800 conjugate secondary antibody (Cat # 611132122, Rockland Immunochemicals Inc.). Blots were visualized and digitally photographed using the Odyssey Infrared Imaging System (model 9120, Li-COR Biosciences, Lincoln, NB, USA).

### Immunoprecipitation

BS-C-1 cells were seeded in 9 × 100 mm tissue culture dishes and grown to 90% confluency (approximately 6.3 × 10^7 ^cells/dish). Cells were infected with a recombinant VACV strain WR vTF7-3 (ATCC VR-2153) expressing a T7 polymerase, at a multiplicity of infection (MOI) of 10, and then transfected with 25 μg ECTV-Moscow 141 (His-tagged) pDEST14 Expression Clone plasmid DNA per 100 mm dish. After 16 h incubation, cells were washed with PBS and lysed with non-denaturing lysis buffer (50 mM Tris-HCl pH 7.5, 300 mM NaCl, 1% Triton ×-100, 10 mM imidazole) containing protease inhibitor cocktail (Cat # 1836153, Roche Applied Science, Indianapolis, IN, USA). After centrifugation at 20,000 ×g for 10 min at 4°C, the lysate supernatant fluid was added to 15 μl Protein G-Plus Agarose (Cat # sc2002, Santa Cruz Biotechnology, Santa Cruz, CA, USA) to pre-clear the extract. Following rotation of the tube at 4°C for 1 h, the Protein G-Plus Agarose was pelleted by centrifuging at 1000 × g for 1 min and Penta-His Antibody (Cat # 34660, mouse Penta-His Antibody IgG_1_, QIAGEN) was added to the supernatant fluid to a final concentration of 5 μg/ml. The tube was rotated for 3 h at 4°C before 60 μl Protein G-Plus Agarose was added and the incubation continued overnight. After ~ 16 h, the Protein G-Plus Agarose was pelleted by centrifuging at 1000 × g for 1 min and the supernatant fluid was removed. The agarose was washed 4 times in 3 ml of wash buffer (0.1% Triton ×-100, 50 mM Tris-HCl, pH 7.5, 300 mM NaCl) and once with PBS. The pellet was resuspended in 80 μl of 1 × NuPAGE LDS sample buffer (Cat # NP0007, Invitrogen Life Technologies) containing 10 mM DTT (dithiothreitol), then heated to 70°C for 10 min and subjected to SDS-PAGE and western blot. The control used herring sperm DNA instead of pDEST14 expression clone DNA.

### Mass spectrometry

Coomassie blue-stained bands were excised from an SDS-PAGE gel using a new scalpel for each band and were prepared and analyzed by the Genome BC Proteomics Centre (Victoria, BC, Canada). The gel slices were subjected to an automated in-gel trypsin digestion, and the proteins obtained were analyzed by MALDI-TOF using a Voyager-DE STR mass spectrometer (Applied Biosystems, Foster City, CA, USA). The Mascot search engine [44] was used to identify the primary protein sequences of the samples from the mass spectrometry data by searching primary sequence databases.

### Far western analysis

2 μg each of porcine muscle tropomyosin (Cat # T2400, Sigma-Aldrich), ECTV-PH (His-tagged, metal chelation chromatography purified), rabbit muscle actin (Cat # A2522, Sigma-Aldrich), RelA (His-tagged bacterial protein, a gift from Dr. Edward Ishiguro, Dept. Biochemistry and Microbiology, University of Victoria), and Bovine Serum Albumin (BSA; Cat # A9647, Sigma-Aldrich) were separated by SDS-PAGE. After transfer to a nitrocellulose membrane, proteins were refolded by a denaturation and renaturation cycle in guanidine hydrochloride as described by Rea *et al*. [45]. The membrane was washed twice in 50 ml of denaturation buffer (6 M guanidine hydrochloride, 20 mM HEPES pH 7.5, 50 mM KCl, 10 mM MgCl_2_, 1 mM DTT, 0.1% Nonidet P-40) for 10 min at 4°C with gentle agitation. The denaturation buffer was diluted 1:1 with basic buffer (20 mM HEPES pH 7.5, 50 mM KCl, 10 mM MgCl_2_, 1 mM DTT, 0.1% Nonidet P-40) and the membrane was washed as before. This dilution and wash cycle was repeated four more times until the final wash contained 175 mM guanidinium hydrochloride. Porcine muscle tropomyosin was used as the probe protein, and was diluted to a final concentration of 20 μg/ml in interaction buffer (1% nonfat dry milk in basic buffer, 5% glycerol, 1 mM PMSF) and was incubated with the membrane for 5 h at 4°C with gentle agitation. The tropomyosin solution was removed and the membrane was washed 4 × 10 min in buffer #1 (0.2% Triton ×-100 in PBS) at 4°C with gentle agitation, followed by 2 × 10 min in buffer #2 (0.2% Triton ×-100, 100 mM KCl in PBS) at 4°C with gentle agitation. The membrane was exposed to mouse monoclonal anti-tropomyosin IgG_1 _primary antibody (Cat # T2780, Sigma-Aldrich) diluted 1:1000 in 1:1 Odyssey Blocking Buffer and PBS + 0.2% TWEEN-20, overnight at 4°C with gentle agitation. The next day the primary antibody was removed and the membrane was washed 4 × 5 min with PBS + 0.1% TWEEN-20. The membrane was then exposed to rabbit anti-mouse IgG (H&L) IRDye 800 conjugate secondary antibody (Cat # 610432020, Rockland Immunochemicals Inc.) at a 1:2500 dilution for 1 h at 4°C with gentle agitation. The secondary antibody was removed and the membrane was washed 4 × 5 min with PBS + 0.1% TWEEN-20 and once with PBS alone. The blot was visualized and digitally photographed using the Odyssey Infrared Imaging System (Li-COR Biosciences).

### Immunofluorescence

BS-C-1 cells were grown on cover slips to 80% confluency, infected with recombinant VACV-WR strain vTF7-3 (ATCC VR-2153; MOI = 10), transfected with 200 ng pDEST14 expression clone plasmid DNA per chamber and incubated for approximately 16 h. After removing growth medium, cells were washed once with RT Tris-buffered saline (TBS; 150 mM Tris-HCl pH 7.4, 150 mM NaCl), fixed for 10 min in 4% paraformaldehyde (in PBS), washed 5 min with TBS, permeabilized in 0.2% Triton ×-100 (in PBS) for 5 min at RT and then washed 3 × 5 min each with TBS. Cells were quenched in fresh 0.1% sodium borohydride (in PBS) for 5 min, washed 3 × 5 min with TBS, blocked (in 10% fetal bovine serum, 1% BSA, 0.02% NaN3, in PBS) for 1 h at RT with gentle agitation and finally washed once for 5 min with TBS.

Cells were incubated in primary antibody diluted in 1% BSA (in TBS) overnight at 4°C with gentle agitation, washed 3 × 5 min with TBS and then incubated with secondary antibody diluted in 1% BSA (in TBS) at RT in the dark for 45 min. Proteins containing a Myc tag were visualized using rabbit polyclonal anti-Myc primary antibody (Cat # 2272, Cell Signalling Technology) 1:100 dilution, and Alexa Fluor 568 conjugate goat anti-rabbit IgG (H+L) secondary antibody (Cat # A11011, Molecular Probes) 1:200 dilution. Proteins containing a HA tag were visualized using Alexa Fluor 488 conjugate mouse monoclonal IgG_1 _anti-HA antibody (Cat # A21287, Molecular Probes) 1:200 dilution. Endogenous cellular tropomyosin was visualized using mouse monoclonal anti-tropomyosin IgG_1 _primary antibody (Cat # T2780, Sigma-Aldrich) 1:200 dilution, and goat anti-mouse IgG (whole molecule) FITC conjugate secondary antibody (Cat # F2012, Sigma-Aldrich) 1:40 dilution. After incubation with secondary antibody, cells were washed 3 × 5 min each with TBS in low lighting, and DNA was visualized by incubation of cells with DAPI (Cat # D5964, Sigma-Aldrich) at 1 ng/ml in TBS for 5 min in the dark. Controls used herring sperm DNA in place of the expression plasmids. After staining with DAPI, cells were washed 3 × 5 min each with TBS in low lighting and coverslips were mounted on slides using the Prolong Antifade Kit (Cat # P7481, Molecular Probes). Pictures of cells were taken at 1000× magnification using the Leica DM6000 B microscope (Leica Microsystems, Richmond Hill, ON, Canada) using the autoexposure option.

## Competing interests

The author(s) declare that they have no competing interests.

## Authors' contributions

CU initiated the project and wrote the manuscript with MJW who also contributed to the bioinformatics analysis. CBC performed all the wet-lab work; MDS performed the structural modelling experiments; GDB performed phylogenetics analysis; RDB helped with immunofluorescence experiments and provided the resources for these experiments. All authors read and approved the final manuscript.
